# Pre-Shell Protection Suppresses Facet-Selective Core Digestion in InP Quantum Dots for High-Yield and Uniform Core/Shell Structures

**DOI:** 10.1021/acsami.5c16396

**Published:** 2025-10-22

**Authors:** You-Cheng Wu, Hsuan-Yu Lee, Hsueh-Shih Chen

**Affiliations:** † Department of Materials Science and Engineering, 34881 National Tsing Hua University, Hsinchu 30013, Taiwan; ‡ College of Semiconductor Research, National Tsing Hua University, Hsinchu 30013, Taiwan; § Department of Chemical Engineering & Materials Science, College of Engineering, Yuan Ze University, Taoyuan 32003, Taiwan

**Keywords:** InP, facet, core/shell, quantum dot, size distribution, digestion, fwhm

## Abstract

Indium phosphide (InP) quantum dots (QDs) are leading cadmium-free alternatives for advanced display technologies, offering wide emission tunability and environmental compliance. However, achieving a high production yield and size uniformity remains challenging. Here we identify a previously overlooked phenomenon, i.e., facet-selective dissolution of InP cores (“core digestion”) during the high-temperature ramping stage in zinc oleate media. This process reduces the number of cores, thereby broadening the core size distribution and disturbing the subsequent shell growth conditions. We introduce a preshell protection strategy that stabilizes the InP core surface before ZnSe/ZnS shell growth, effectively suppressing core digestion. As a result, the obtained core/shell QDs exhibit an enhanced particle yield, improved size uniformity, and superior optical performance. This work reveals a degradation pathway in InP synthesis and provides a practical route to scalable, high-quality QDs for display applications.

## Graphical abstract

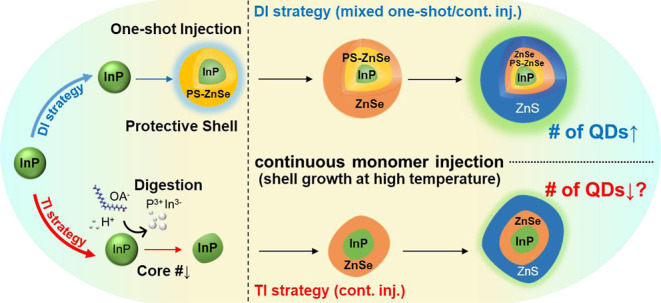

## Introduction

Colloidal quantum dots (QDs) are highly promising nanomaterials due to their exceptional optical properties, including a narrow full width at half-maximum (fwhm), high photoluminescence (PL) quantum yield (QY), and tunable bandgap. With these properties, QDs can be applied in many fields, such as solar cells, biolabeling, photodetectors, and displays.^1–10^ For the display, despite the outstanding optical characteristics of Cd-based QDs,^11–13^ their practical application has been impeded by the presence of toxic heavy metals, prompting a significant demand for safer alternatives. Indium phosphide (InP) QDs exhibit remarkable optical properties and are characterized by their nontoxic nature. These unique attributes position them as promising alternative materials to Cd-based QDs for display applications.^4,14–17^

In recent years, researchers have made significant efforts to optimize the optical performance of InP QDs. The prevailing approach involves thick shell epitaxial growth of the inner ZnSe shell and outer ZnS shell on InP cores to enhance the optical properties and stability.^18–23^ For the growth of ZnSe and ZnS, the commonly used precursor is zinc oleate (ZnOA_2_).^19,24–27^ To improve the shell crystal structure, the high shell growth temperature (>280 °C) has been adopted.^28,29^ This suggests that the InP cores were subjected to a process involving their mixing with zinc oleate at an elevated temperature. However, previous studies have explored the etch effects of ZnOA_2_ on InP QDs at room temperature, resulting in alterations to the crystal structure and size of InP QDs.^30^ In the shell growth process, much higher concentrations of ZnOA_2_ and the elevated temperature can exert a more substantial influence on the InP cores, which would impact the ultimate optical properties and quantity of InP/ZnSe/ZnS core/shell QDs.

In this study, we report a previously unrecognized phenomenon: the dissolution of QD cores occurring prior to shell growth at elevated temperatures, significantly influencing both the yield and the performance of core/shell QDs. This previously overlooked core dissolution, triggered by ZnOA_2_, reduces the QD number and compromises product uniformity. To mitigate core dissolution, we implemented a strategic preinjection of shell monomers during the dissolution phase, initiating the growth of a preliminary ZnSe shell as protection against core dissolution. This preshell effectively protects the InP cores during temperature ramp-up in the ZnOA_2_ solution, maintaining their crystallinity and integrity. As a result of this protective shell approach, the number yield of InP/ZnSe/ZnS QDs increased substantially, approximately 204% in 10-*g*-scale synthesis. The resulting QDs demonstrate exceptional optical properties, achieving a PL QY of nearly 100% and a fwhm of 37 nm.

## Experimental Section

### Chemicals

Indium acetate (In­(OAc)_3_, 99.9%), zinc acetate (Zn­(OAc)_2_, 99.9%), oleic acid (OA, 90%), octadecene (ODE, 90%), tris­(trimethylsilyl)­phosphine ((TMS)_3_P, 20% in ODE) and trioctylphosphine (TOP, 90%), selenium powder (Se powder 99.99%), sulfur powder (S powder, 99.5%), hydrofluoric acid (HF, 49%), toluene (>99.8%), and acetone (>99%) were used as received.

### Preparation of 0.4 M Zn­(OA)_2_ Stock Solution

120 mmol portion of Zn­(OAc)_2_ and 240 mmol of OA were combined in 225 mL of ODE within a 500 mL three-neck flask. The flask was heated to 150 °C under vacuum until the turbid solution turned transparent, and subsequently cooled to room temperature.

### Preparation of 0.5 M TOPSe

A 25 mmol portion of selenium powder and 50 mL of TOP were heated at 80 °C until transparent and then cooled to room temperature.

### Preparation of 0.5 M TOPS

A 25 mmol portion of sulfur powder and 50 mL of TOP were heated at 80 °C until transparent and then cooled to room temperature.

### Synthesis of InP Cores

One millimole of In­(OAc)_3_, 1 mmol of Zn­(OAc)_2_, and 4 mmol of OA were mixed in 14.65 mL of ODE in a 100 mL three-neck flask. The mixer was heated to 150 °C under vacuum until the turbid solution changed to transparent, and then the solution was cooled to 30–32 °C. The flask was filled with N_2_, and 0.75 mmol (TMS)_3_P and 8 mL of TOP were injected into the solution. Following the injection, the solution was heated to 305 °C (20 °C/min) and kept for 11.5 min. The heating mantle was removed to cool the core solution. The quantity or particle number of InP cores was calculated according to the Beer–Lamber equation, which is listed in the Supporting Information (Figure S2).

### Purification of InP Cores

To remove residuals, 25 mL of core solution was precipitated by adding 50 mL of toluene, 275 mL of acetone, and 25 mL of methanol and centrifuged at 4000 rpm for 40 min. After purification, the InP core was redispersed in 12 mL of ODE.

### Synthesis of InP/ZnSe/ZnS QDs

Nine milliliter of 0.4 M Zn­(OA)_2_ stock solution was heated to 150 °C in a 50 mL three-neck flask, and 3 mL of purified core (estimated ∼3.6 × 10^–7^ mol) was injected into the solution. The flask was filled with N_2_, and then 10 μL of HF with 40 μL of acetone was injected into the mixture. For the dual injection (DI) strategy, 1.4 mL of 0.5 M TOPSe was injected into the solution at 150 °C. (For the traditional injection (TI) strategy, the temperature was heated to 320 °C directly without 1.4 mL of 0.5 M TOPSe being injected.) After the injection, the solution was heated to 320 °C. During the heating-up process, the syringe pump containing 0.5 M TOPSe was activated at 280 °C (0.6 mL/h) (for the TI strategy, the injection rate of 0.5 M TOPSe was 2 mL/h). After 60 min, the ZnSe shell growth was completed, and the syringe pump containing 0.5 M TOPS was activated for 150 min (1.2 mL/h) to form the ZnS shell.

### Digestion Experiment of InP Cores

For the digestion experiment of InP cores in a ZnOA_2_ solution, 9 mL of 0.4 M ZnOA_2_ and 1 mL of ODE solution were mixed in a 50 mL three-neck flask. The mixture solution was kept under a vacuum and heated to 150 °C for 1 h. The atmosphere was then switched to nitrogen. Subsequently, 2 mL of purified InP core solution in the ODE was injected, and the temperature was elevated to 320 °C. Aliquots were taken at 150 °C, 280 °C, and 320 °C for UV–vis measurements at room temperature. For the digestion experiment of the InP core in the ODE solution and OA solution, the procedure was the same as described above, but the solvent was changed to 10 mL of the ODE solution, 2.27 mL of the OA solution, and 7.73 mL of the ODE solution.

### Characterization

UV–visible absorption measurements were conducted using a Jasco V-770 spectrophotometer. (PL) spectra was recorded using an Edinburgh FS5 (SC-05) spectrofluorometer. The PLQY was determined through absolute QY measurements in an integrating sphere calibrated by standard phosphors utilizing an Edinburgh FS5 (SC-30) spectrometer. For the above measurement, QDs dissolved in toluene were contained within a quartz cell, ensuring an absorbance value below 0.2 to minimize the impact of the QD concentration. The UV–vis and PL spectra were acquired at room temperature. High-resolution transmission electron microscopy images were captured by using a JEOL JEM-F200 operating at 200 keV, with the QDs deposited onto copper grids. X-ray diffraction (XRD) measurements were performed by using a D2 PHASER X-ray diffractometer operating at 30 kV/10 mA, utilizing the Cu Kα line (λ = 1.54184 Å) with QDs deposited on silicon substrates. Energy dispersive X-ray spectroscopy (EDS) analysis was carried out using an FESEM-8010 scanning electron microscopy system with the QDs deposited on glass substrates. Time-resolved photoluminescence (TRPL) spectra were recorded using a PicoHarp300 with time-tagged time-resolved (TTTR) Mode and a PHR 800 router (PicoQuant, Berlin, Germany), and the wavelength of the excitation laser was 405 nm. Single-photon blinking measurements of the QDs were performed using a self-modified confocal microscope (SouthPort Co.). The QDs were mixed with poly­(methyl methacrylate) (the molecular mass was 350,000) and spin-coated onto the cover glass. The well-dispersed QD samples were excited by a single-mode 405 nm pulsed diode laser (LDH-P-C-405B, PicoQuant) and a CW laser (Civil Laser) with an oil-immersion objective lens (UPlanSApo, NA = 1.4, Olympus). The lasers were focused to a spot size of about the diffraction limit (diameter ∼353 nm) on the samples. The PL signals were sent to avalanche photodiodes (SPCM-AQRH-16, Excelitas) connected to a TTTR module (HydraHarp 400, PicoQuant).

## Results and Discussion

### Occurrence of Dissolution of InP Cores Prior to the Shell Growth

In conventional two-step synthesis methods, InP cores are initially prepared in a smaller reactor and subsequently overcoated with a ZnS shell in a larger reactor. Typically, OA serves as the shell growth medium by dissolving Zn salts to form ZnOA_2_, acting as Zn precursors. In our previous studies involving the synthesis of large quantities (>10 g) of InP/ZnSeS–ZnS QDs, we noted that both shell thickness and particle size distribution were influenced by the heating rate of the growth medium (data not shown). Additionally, during shell formation, a decrease in optical absorbance of InP/ZnSeS QDs over time indicated a reduction in particle number. The final size distribution of QDs was also affected by the duration that cores spent in a high-concentration metal oleate solution at elevated temperatures. A control experiment demonstrated a significant reduction in the particle number of InP cores after heating in a metal oleate solution without introducing S/Se precursors, confirming previous observations that metal oleates etch InP cores.^31^ Thus, core etching by the metal oleate growth medium during shell formation is identified as a key factor contributing to the broader size distribution and lower yield of the final QD product.

To investigate the etching effect of the shell precursor ZnOA_2_ on InP cores, we directly annealed InP cores in different media (OA or ZnOA_2_) without chalcogen (S or Se) monomer injections. Three solvent media commonly used for QD shell growth, e.g., ODE, ZnOA_2_ dissolved in the ODE, and OA dissolved in the ODE, were evaluated for their ability to etch InP cores. The InP cores utilized in this study exhibited a first excitonic optical absorption peak at 465 nm, corresponding to a diameter of approximately 1.8 nm, as confirmed by optical absorption measurements and TEM imaging (Figure S1). EDS analysis indicated that these InP cores possess an atomic ratio of In/P/Zn approximately equal to 40:50:10 (Figure S3). The cores were introduced into each selected solvent medium at 150 °C and then heated to 320 °C, simulating the temperature profile typically encountered during the shell growth process.

Figure 1 gives the optical absorption spectra and estimated particle concentrations of InP cores annealed in different solutions. As-prepared InP cores exhibit the first exciton optical absorption at 465 nm and have a diameter of 1.8 nm (Figure S1). As they are annealed in pure ODE at ∼320 °C (bp = 315–320 °C), the absorption peak of InP cores slightly redshifts from 465 nm (sample collected at *T* = 150 °C) to 467 nm (*T* = 280 °C) and 469 nm (*T* = 320 °C), respectively, implying a slight increase in the core size. It is also noted that the optical absorbance of the first exciton absorption peak slightly broadens, and the value decreases from 0.225 (λ = 465 nm, sample collected at 150 °C) to 0.160 (λ = 469 nm, sample collected at 320 °C) after the heating process, suggesting a decreased number of InP cores. Moreover, in ZnOA_2_/ODE, the InP absorption peak blueshifts ∼15 nm from 465 nm (*T* = 150 °C) to 450 nm (*T* = 280 °C), suggesting decreased core size. As temperature increased to 320 °C, the peak redshifts ∼24 nm back to 474 nm due to Zn passivation on the InP core surface.^9,31,32^ The optical absorbance significantly decreases from 0.225 (λ = 465 nm, *T* = 150 °C) to 0.022 (λ = 474 nm, *T* = 320 °C), and the absorption peak becomes more broadening after the annealing process. This suggests that incorporation of ZnOA_2_ could decrease a larger amount of InP cores. To investigate the role of oleates, InP cores are directly annealed in pure OA with ODE. It is found that the absorption peak significantly blueshifts ∼24 to 441 nm (*T* = 150 °C) compared with those annealed in either ODE or ZnOA_2_/ODE. The optical absorbance also dramatically decreases from 0.225 (as-prepared before heating) to roughly ∼0.1 (*T* = 320 °C). Also, it can be observed that the reaction mixture turns transparent by naked eyes. Figure 1d presents the variation of the particle concentration of InP cores calculated by the Beer–Lamber equation (Supporting Information).^33^ It exhibits that the particle number of InP cores heated in pure ODE medium slightly decreases with increasing temperature, that is, 89% at 280 °C and 71% at 320 °C of the initial value. In contrast, the particle number of InP cores significantly decreases in both ZnOA_2_ and OA media during the heating process. In ZnOA_2_ solvent, the particle number drops to 49% and 10% at 280 and 320 °C, respectively. The event is much more severe in OA. InP cores nearly disappear when heated at more than 280 °C. These dramatic dissolution effects can be rationalized by considering the ligand reactivity and acidity of the medium. OA contributes protons that may react with surface In^3+^, leading to the formation of soluble indium oleate complexes (In­(OA)_3_). Meanwhile, Zn­(OA)_2_ provides both oleate anions and Zn^2+^ ions, and the oleate anions could coordinate with surface atoms as Lewis bases, facilitating lattice detachment, while Zn^2+^ ions can displace indium carboxylates on the InP surface.^34,35^ This distinction emphasizes that the behavior of OA is not the same as that of the oleate species from ZnOA_2_. In contrast, the ODE is relatively inert and lacks coordinating species, thereby preserving the core integrity.

**Figure 1:**
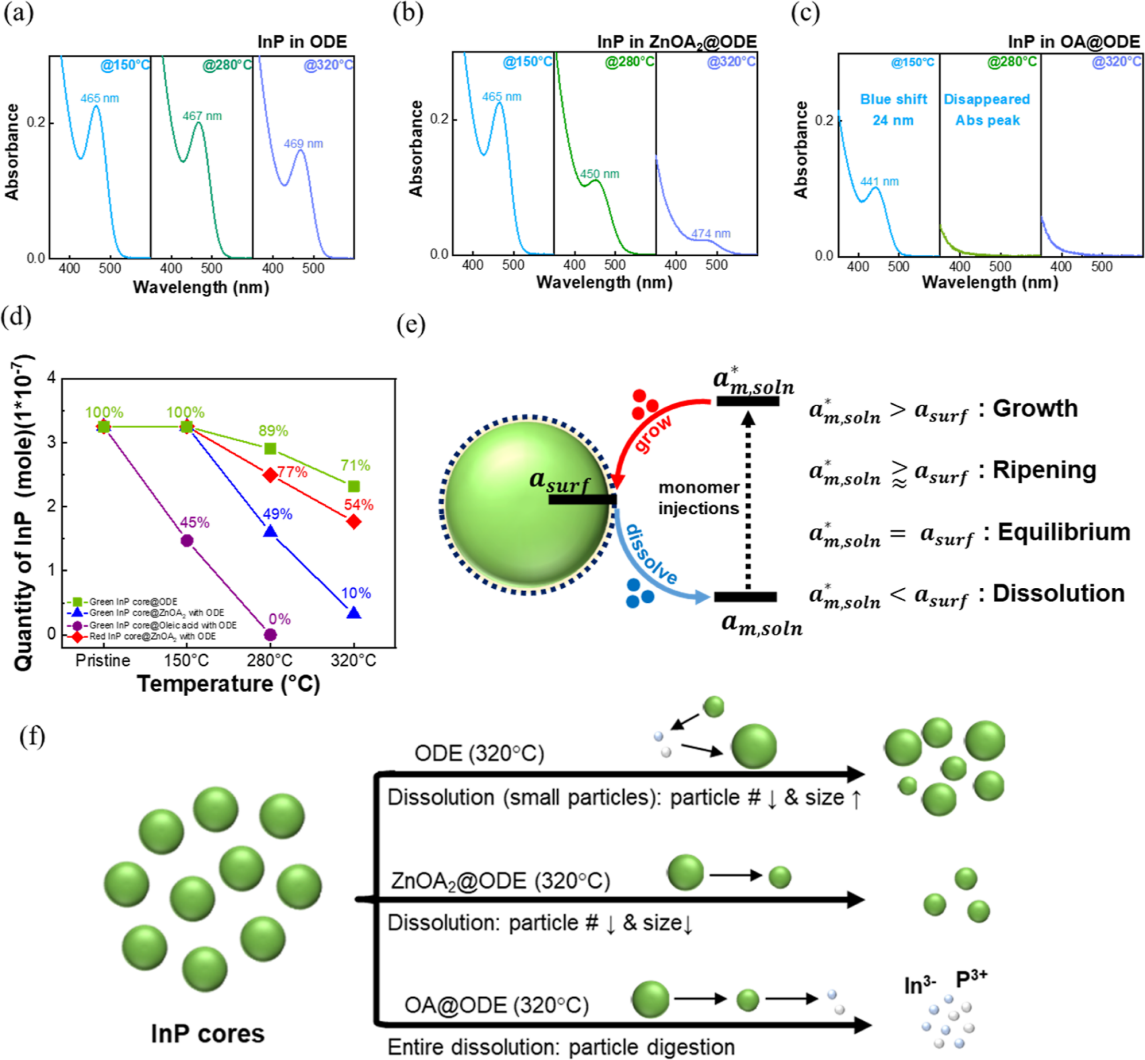
Variation of optical absorption spectra of InP cores collected at 150 °C, 280 °C, and 320 °C when annealing in different solvent media without S-monomer injection. Samples were without any purification. (a) InP cores heated in an ODE solution. (b) ZnOA_2_ with ODE solution and (c) OA with ODE solution. (d) The variation in the quantity of InP core during the heating process from 150 to 320 °C in ODE, ZnOA_2_ with ODE, and OA with ODE solution. During heating, a fixed aliquot of the reaction mixture was taken from the reactor and was immediately quenched in toluene for optical absorption measurements. The absorption was monitored at the initial peak position to ensure the same. (e) Schematic presentation of thermodynamic growth events for a QD nanocrystal. The monomer activity at the QD surface (***a***_**m,surf**_) and in the solution (***a***_**m,soln**_). After extra monomer injections, the activity increases (***a***_m,soln_*). (f) Schematic illustration of InP cores annealing in different solution media at 320 °C. The boiling point of ODE is 315–320 °C.

The phenomenon observed in the present study is referred to as the “digestion” of InP cores. The growth behavior of QD nanocrystals is governed by the monomer activity (***a***_**m**_) at the nanocrystal surface (***a***_**m,surf**_) relative to that in the solution medium (*a*_*m*,soln_). Introducing additional monomers can elevate the solution monomer activity (***a***_m,soln_*), initiating a monomer diffusion process. Depending on the relative magnitudes of monomer activity, crystal growth occurs when the overall solution monomer activity surpasses the surface monomer activity (***a***_m,soln_* > ***a***_**m,surf**_), while dissolution occurs when it is lower (***a***_m,soln_* < ***a***_**m,surf**_), as illustrated in Figure 1e. A ripening process generally occurs near thermodynamic equilibrium when the solution monomer activity is slightly higher than or nearly equal to the QD surface activity (***a***_m,soln_* > ***a***_**m,surf**_), whereas exact equality (***a***_m,soln_* = ***a***_**m,surf**_) indicates equilibrium.

Figure 1f schematically illustrates three different dissolution events of InP cores in different solvent media proposed in the present study. These schematic comparisons support the interpretation that ligand strength and medium acidity govern the degree of InP core digestion. The occurrence of the core digestion event is proposed to be driven by thermodynamic equilibrium between the InP core surface and the reaction medium. When the InP cores are dispersed in a solvent medium such as Zn­(OA)_2_/ODE or OA/ODE, followed by heating at a relatively high temperature, dissolution of surface atoms or lattices of the InP cores would occur to a certain extent. In a medium with reactive molecules such as OA or oleates, dissolution would spontaneously happen. For example, OA could provide protons to dissolve the InP surface and form a metal oleate In­(OA)_3_.^31,32,36,37^ In addition, the digestion event should be distinguished from Ostwald ripening,^4,38^ where smaller crystals dissolve back to monomers that supply the continued growth of larger crystals near the thermodynamic equilibrium, while in the present study, the digestion happens because of solution etching during the QD shell growth.

### Dissolution of InP Cores during Shell Growth and Proposed Strategy

Shell growth is typically performed at temperatures exceeding 280 °C.^30,39^ Based on our observations, InP cores can undergo digestion during the temperature ramping phase, leading to a significant reduction in the core quantity at higher temperatures before shell precursor injections. Preventing this core digestion is crucial, as it increases the uncertainty in shell growth and negatively impacts both the production yield and the luminescence efficiency of the resulting QDs. Core digestion arises from surface dissolution, which is size- and temperature-dependent due to variations in crystal surface solubility. Thus, instead of maintaining a constant shell monomer concentration, it is preferable to adjust the monomer concentration throughout the shell growth process. For uniform shell formation, shell monomers must be evenly deposited onto each core lattice, a process strongly influenced by the thermodynamic activity between the core surfaces and the monomers in solution. However, maintaining consistent thermodynamic activity during shell growth is challenging, as both the core surface and monomer activities fluctuate with temperature and solution composition. Therefore, we propose a combined injection strategy comprising an initial low-temperature pulse injection of monomers followed by a continuous injection at higher temperatures. The initial pulse injection provides an adequate monomer concentration during the temperature ramping stage, effectively preventing core dissolution. Subsequently, the continuous injection at elevated temperatures ensures a sufficient monomer supply for uniform shell formation. In addition, the concentration of injected Zn and Se monomers was estimated to be less than 30% of the critical level required for independent nucleation, ensuring that growth occurred exclusively on the preformed InP seeds. Figure 2 schematically compares the growth of core/shell QDs using the TI strategy and the proposed mixed pulse/continuous DI strategy. In the TI strategy, InP cores are susceptible to dissolution (designated as ZnOA_2_–InP) prior to shell growth.

**Figure 2:**
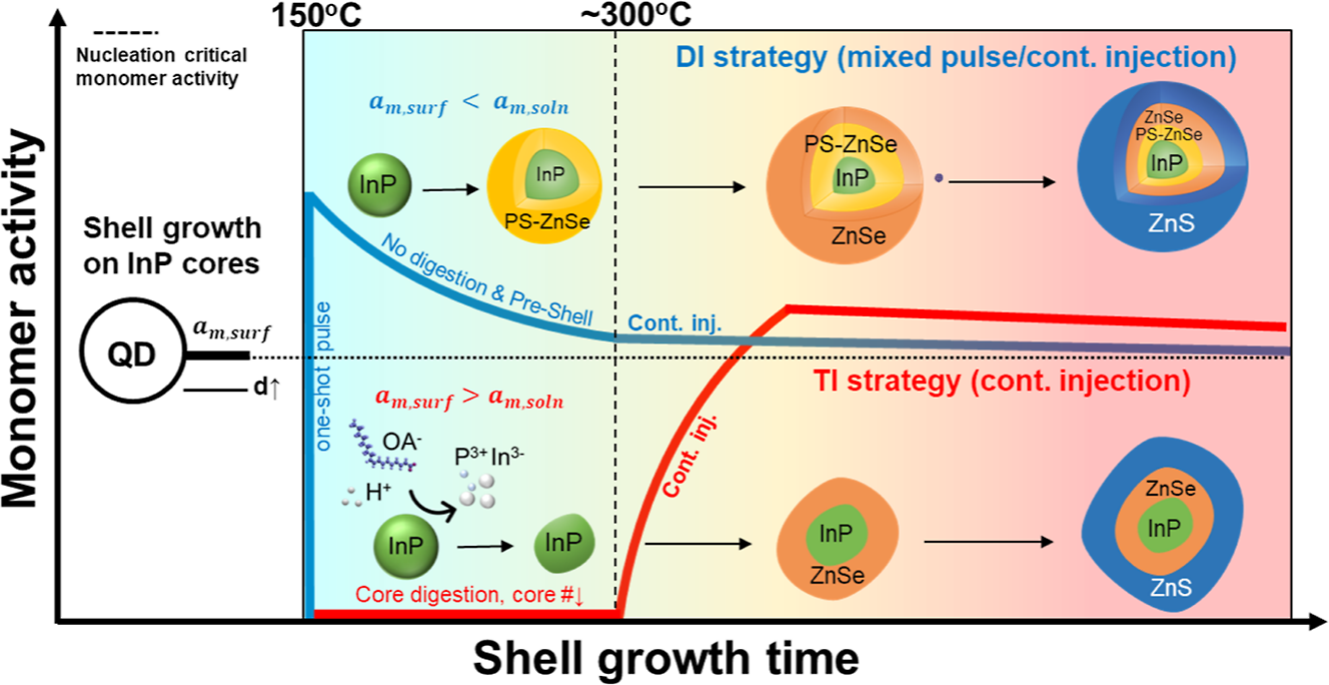
Schematic illustration comparing monomer activity variations of InP/ZnSe/ZnS core/shell QDs under two distinct growth strategies. In the TI method, InP cores within the growth medium undergo digestion prior to shell monomer injections. This digestion leads to particle number loss and uneven surface morphology. In the modified DI method, a one-shot pulse preinjection of monomers to grow a preshell to prevent digestion of the cores. The preinjection of ZnSe monomers was controlled below 30% of the critical concentration of nucleation.

XRD analysis (Figure 3a) reveals that the core dissolution predominantly occurs at the (220) and (311) lattice planes (ZnOA_2_–InP, red curve), as evidenced by the significant reduction in diffraction intensities for these planes, leaving only the primary (111) peak. This also implies a preferential attack or etching of the InP (220) and (311) facets by environmental species. Similar facet-selective behaviors have been previously observed in Cd-based QDs, where certain facets such as (100) were preferentially doped or oxidized into ZnSO_4_/Zn­(OH)_2_ (002) structures.^40–42^ By an initial pulse injection of shell monomers to grow a preliminary shell, the InP cores can be protected against digestion (PS-InP, green curve).

**Figure 3:**
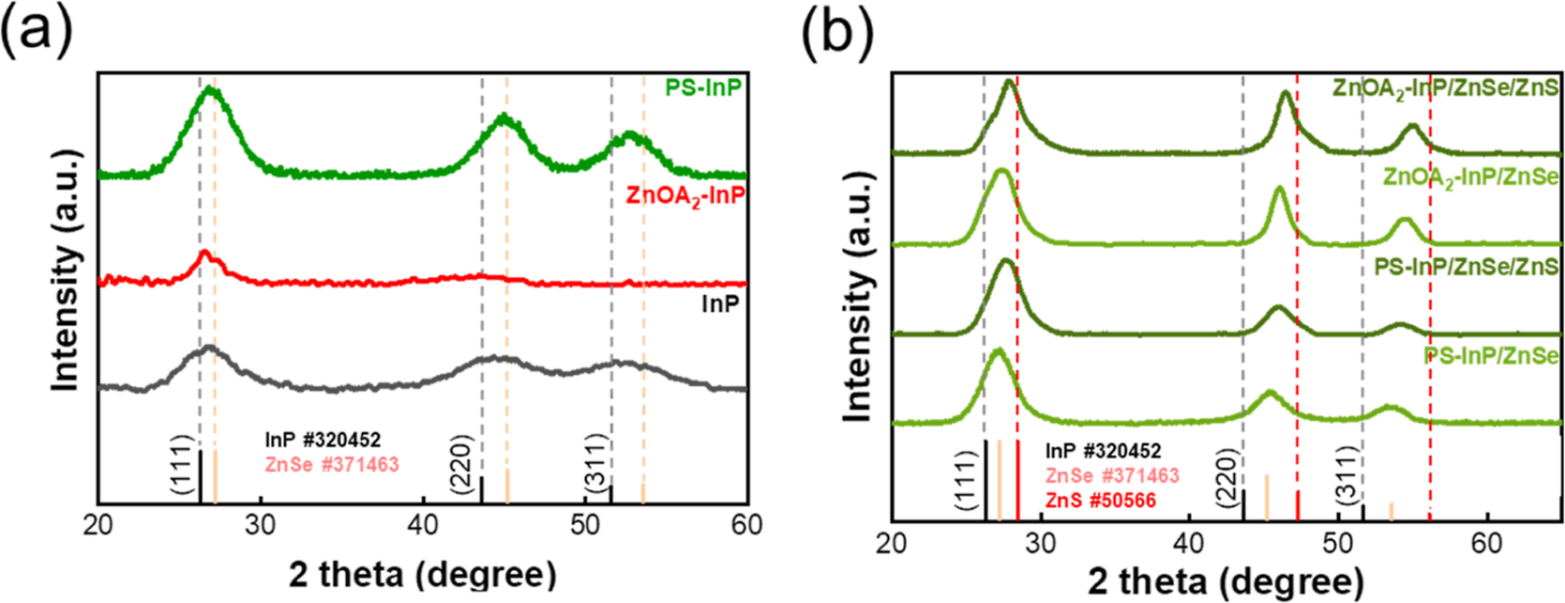
Effect of ZnOA_2_ etching on the structure and composition changes of InP cores and following ZnSe/ZnS shell growth. (a) XRD patterns of as-prepared InP cores (InP, black), ZnOA_2_-treated InP cores (ZnOA_2_–InP, red curve), and InP core with preliminary ZnSe shell (PS-InP, green curve) grown by a pulse injection before the formal ZnSe/ZnS shell growth. (b) XRD patterns of PS-InP and ZnOA_2_–InP cores further grown with inner ZnSe shell and other ZnS shell overcoating. Both PS-InP and ZnOA_2_–InP exhibit diffraction peaks shifting to a higher angle after the shell growth due to the smaller lattice constant of ZnSe (5.67 Å) and ZnS (5.41 Å) compared to InP (5.87 Å). ZnOA_2_–InP shows a more significant shift in diffraction peaks, which is induced by the uncontrollable thicker shell thickness. The average size of PS-InP is estimated ∼4.6 nm, indicating that the thickness of ZnSe preliminary shell is ∼2.8 nm (core size ∼1.8 nm, shown in Figure S1).

The impact of core digestion on subsequent shell growth is shown by XRD patterns (Figure 3b). After grown with inner shell ZnSe and outer ZnS shell, the (220)/(311) XRD peaks of ZnOA_2_-etched InP cores (ZnOA_2_–InP/ZnSe and ZnOA_2_–InP/ZnSe/ZnS) obviously become more intensive compared with PS-InP cores (PS-InP/ZnSe and PS-InP/ZnSe/ZnS), implying that the core etching leads to anisotropy shell growth. This intensity increase likely originates from anisotropic overgrowth of shell material along the etched facets, rather than a uniform core/shell structure. This highlights how the core structure and composition inherently affect subsequent shell growth and the final core/shell morphology.

In addition, it is worth noting that when the particle number of InP cores decreases due to digestion, shell growth may occur at a relatively higher monomer concentration. Furthermore, the shell growth rate may vary for digested cores of different sizes, which easily leads to uncontrollable results and potentially results in the formation of some large core/shell particles with relatively lower PLQY between 40% and 60% (Figure S5). These oversized particles are relatively scarce and are typically removed during subsequent purification steps, for example, as part of the precipitate collected after high-speed centrifugation, and can be readily identified under UV illumination.

Moreover, the PLQY of the QD samples is generally measured in dilute colloidal solutions with an optical density below 0.1, where oversized particles are minimal or effectively excluded through purification. As a result, such measurements may not fully reflect the particle-level heterogeneity or the presence of nonemissive species in the entire product batch. In contrast, the DI strategy involves a pulse monomer preinjection, referring to a one-time Se monomer supply during ramping, which leads to the formation of a preliminary ZnSe shell (PS) on the InP cores (designated as PS-InP), serving as a protective layer.^31^ TEM analysis indicates that PS-InP cores have an increased diameter of approximately 4.6 nm (Figure S4), corresponding to a PS thickness of roughly 1.4 nm. XRD results further demonstrate that the (220)/(311) diffraction peaks of the PS-InP QDs remain robust with increased intensity, confirming that the epitaxially grown ZnSe preliminary shell effectively protects the InP cores from dissolution. This indicates that the pulse preinjection or ZnSe preshell preferentially stabilizes the more vulnerable (220) and (311) planes, which are otherwise etched in the absence of protection.

### Comparative Study of Optical Properties of InP/ZnSe–ZnS QDs Synthesized via TI vs DI Strategies

Figure 4 presents the optical absorption and PL spectra of PS-InP and ZnOA_2_–InP cores during the shell growth process. The ZnOA_2_–InP cores exhibit a noticeable blue-shift of 16 nm due to core digestion. After epitaxial growth of ZnSe and ZnS shells, the PS-InP/ZnSe–ZnS QDs demonstrate an PLQY of 92% and a fwhm of 37 nm. In contrast, the ZnOA_2_–InP/ZnSe–ZnS QDs show a lower PLQY of 64%. These results indicate that digestion of InP cores not only reduces the quantity of cores but also influences the optical performance of the resulting core/shell QDs since the growth condition could be changed.

**Figure 4:**
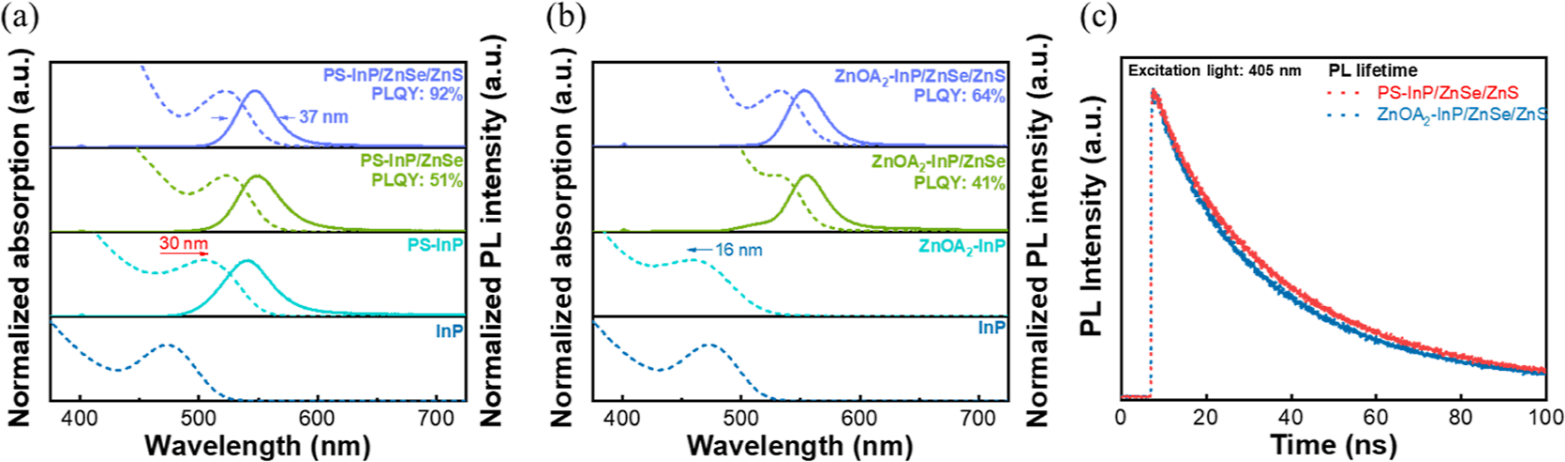
Evolutions of optical absorption (dashed line) and PL (solid line) spectra of core/shell QDs grown from as-prepared InP cores during the shell growth. Samples were collected from the reaction mixtures. (a) PS-InP (ZnSe-preshelled InP cores after a pulse Se monomer injection), PS-InP/ZnSe (PS-InP further overcoated by ZnSe shell after continuous TOP-Se injection), and PS-InP/ZnSe/ZnS (PS-InP/ZnSe further overcoated by ZnS shell). (b) ZnOA_2_–InP during shell growth process. (c) Time-resolved PL (TRPL) spectra of PS-InP/ZnSe/ZnS and ZnOA_2_–InP/ZnSe/ZnS QDs.

To further explore the differences in optical properties, we conducted an analysis of the PL decay for PS-InP/ZnSe–ZnS and ZnOA_2_–InP/ZnSe–ZnS, as depicted in Figure 4c. The PL decay curves for both InP core/shell QDs can be fitted by a triexponential function, that is, *I*(*t*) = *A*_1_ exp­(−*t*/τ_1_) + *A*_2_ exp­(−*t*/τ_2_) + *A*_3_ exp­(−*t*/τ_3_), where *I* is the PL intensity at a specific time (*t*), *A* is the weight constant with *A*_1_ + *A*_2_ + *A*_3_ = 1, and τ is the decay time constant. Values of each parameter can be found in Table S1. The fitted components reflect different recombination pathways. τ_1_ (∼8 ns) corresponds to fast, nonradiative recombination caused by surface-related defects such as oxides or unpassivated atoms. τ_2_ (∼20–40 ns) represents radiative exciton recombination through the InP band-edge and serves as the main contributor to PL emission. τ_3_ (∼111 ns) is attributed to deep trap-related states, typically associated with crystal imperfections or midgap states, which can significantly lower PLQY.^43–46^ τ_2_ corresponds to band-edge emission and serves as the primary radiative recombination channel.^28,47,48^ Compared with ZnOA_2_–InP/ZnSe/ZnS, PS-InP/ZnSe/ZnS QDs have a relatively larger *A*_2_ (0.79 > 0.73) and a lower value of defect-related components. These differences suggest that the DI-prepared QDs possess fewer nonradiative recombination centers and more effective band-edge emission pathways. The reduced contributions of the τ_1_ and τ_3_ components imply better surface passivation and fewer deep-level traps, which can reduce the probability of trap-mediated recombination and intermittent luminescence. The observed PL decay results are in line with a high PLQY for PS-InP/ZnSe/ZnS QDs.

### Effect of Digestion on the PLQY of QDs and Light-Conversion Efficiency of QD Color Conversion Films

Due to the digestion of InP cores, their quantity significantly decreases before shell growth, impacting subsequent shell deposition conditions. To further examine how core digestion influences the final QD products, size variations of PS-InP cores (prepared by the DI method) and ZnOA_2_–InP cores (prepared by the TI method), each overcoated with ZnSe/ZnS shells under identical experimental conditions, are analyzed using TEM, as presented in Figure 5. The PS-InP cores initially measuring 4.6 nm increased to an average size of 6.6 ± 0.7 nm after ZnSe shell deposition (PS-InP/ZnSe) and further expanded to 9.0 ± 1.0 nm upon an additional ZnS coating (PS-InP/ZnSe/ZnS). In contrast, ZnOA_2_–InP cores prepared with the TI strategy show a larger average size increase, reaching 10.7 ± 1.5 nm after ZnSe shell growth (ZnOA_2_–InP/ZnSe) and further enlarging to 13.3 ± 1.6 nm after subsequent ZnS coating (ZnOA_2_–InP/ZnSe/ZnS) (Figure S6). These differences result from monomer overdeposition on fewer surviving cores in the TI method, which accelerates shell growth and leads to nonuniformity and lower optical performance.

**Figure 5:**
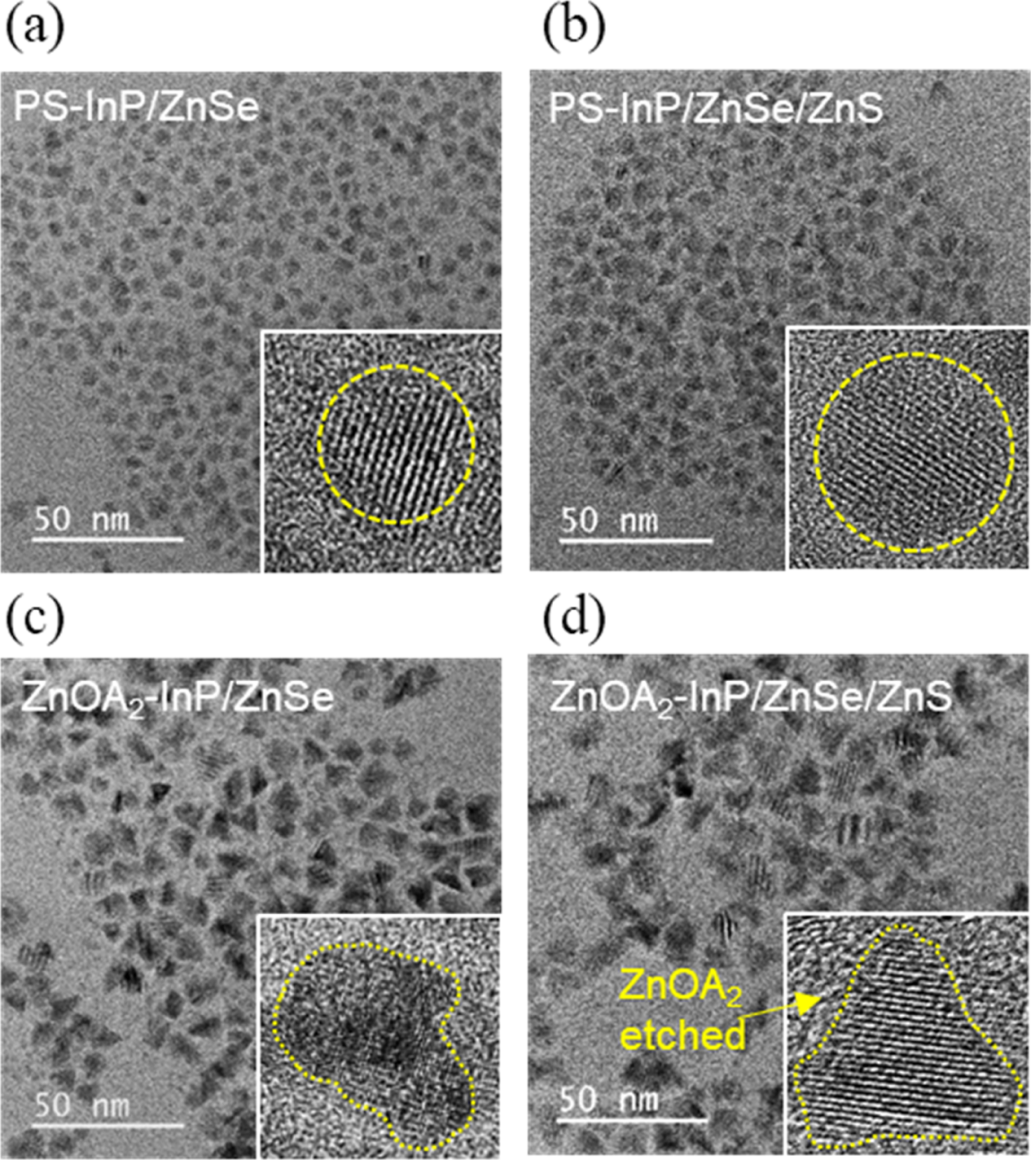
TEM images of QDs grown with (PS-InP) and without protective shell (ZnOA_2_–InP). Low resolution image and a selected QD in higher resolution (inset) of PS-InP cores overcoated with inner ZnSe shell (PS-InP/ZnSe, *d* = 6.6 ± 0.7 nm, σ = 9.9%) (a) and outer shell (PS-InP/ZnSe/ZnS, *d* = 9.0 ± 1.0 nm, σ = 10.9%) (b). ZnOA_2_-InP cores overcoated with inner ZnSe (ZnOA_2_-InP/ZnSe, *d* = 10.7 ± 1.5 nm, σ = 14.4%) (c) and outer ZnS shell (ZnOA_2_–InP/ZnSe/ZnS, *d* = 13.3 ± 1.6 nm, σ = 12.2%) (d). The size histograms can be found in Figure S6.

Evidently, core digestion significantly influences the shell thickness and size distribution of the final QD products even when synthesized under identical initial conditions. In the TI method, InP cores directly exposed to the reaction solvent medium are prone to digestion during shell growth. Consequently, the reduced number of surviving cores undergoes shell overcoating at relatively higher shell monomer activity because fewer cores remain to consume the shell monomers, potentially explaining why ZnOA_2_–InP/ZnSe/ZnS QDs prepared via the TI method exhibit larger average sizes (∼13.3 nm), greater size deviation, and lower PLQY ∼64%.^29,49^ Additionally, core digestion is sensitive to the temperature ramp rate, leading to unpredictable shell growth outcomes. Although the “weight yield” of the final products from both DI and TI methods appears similar due to the redeposition of dissolved monomers onto remaining large particles, the particle number yield is significantly lower in the TI method. This reduction in QD number leads to fewer emitters in QDCC films and, consequently, a reduced LCE.

Even when the product contains a mixture of good and defective QDs, postpurification measurements can still report high PLQY values due to the preferential retention of bright particles. However, this can be misleading because the QD optical properties are usually measured under dilute conditions, where a small number of low-PLQY or oversized particles might be overlooked or even removed during purification. As a result, the measured PLQY may overestimate the true quality and uniformity of the product. The key difference between the TI and DI methods lies not in the individual brightness of QDs but in the total number of well-performing particles produced. If the purification process fails to remove larger or defective QDs, then the final product may still contain heavy, low-efficiency particles. When this happens, even if the total weight of QDs is the same, fewer bright and uniform QDs are actually present in the polymer matrix. This reduces the number of emitters per volume and lowers the light-conversion efficiency (LCE) in the QDCC films.

Figure 6a,c presents the single-photon fluorescence intensity time traces for PS-InP/ZnSe/ZnS and ZnOA_2_–InP/ZnSe/ZnS. QD blinking is characterized by random fluctuations between a bright (on) state and a dark (off) state. Frequent blinking or prolonged residence in the dark state can significantly influence the overall PL intensity of the QD ensemble.^50,51^ In the obtained results, PL intensities above the dashed line indicate the on-state, while those below correspond to the off-state. The histograms in Figure 6b,d represent the statistical proportions of on and off states based on 50 measurement results. Through statistical analysis, it is observed that PS-InP/ZnSe/ZnS exhibits an average on-state proportion of approximately 64%, whereas ZnOA_2_–InP/ZnSe/ZnS shows only about 24%. This suggests that for most of the time, a single QD of ZnOA_2_–InP/ZnSe/ZnS does not emit photons and exhibits a higher blinking frequency. The occurrence of the blinking phenomenon is associated with Auger recombination and trap states.^52^ In the case of Auger recombination, when an additional electron or hole is present, the excess energy of a higher-energy electron may be transferred to a third particle instead of being released as a photon, resulting in a nonradiative recombination pathway. Similarly, if trap states are present, excitons can be captured, generally leading to nonradiative recombination. Both mechanisms can coexist, contributing to the observed blinking behavior. For ZnOA_2_–InP/ZnSe/ZnS, the higher blinking frequency implies that nonradiative pathways are more prevalent, leading to an increased proportion of the off-state in single-photon QDs. These nonradiative pathways may be linked to the core digestion event during the shell growth, possibly increasing the probability of the formations of In–S bonds and Se–Se, which introduce additional trap states.

**Figure 6:**
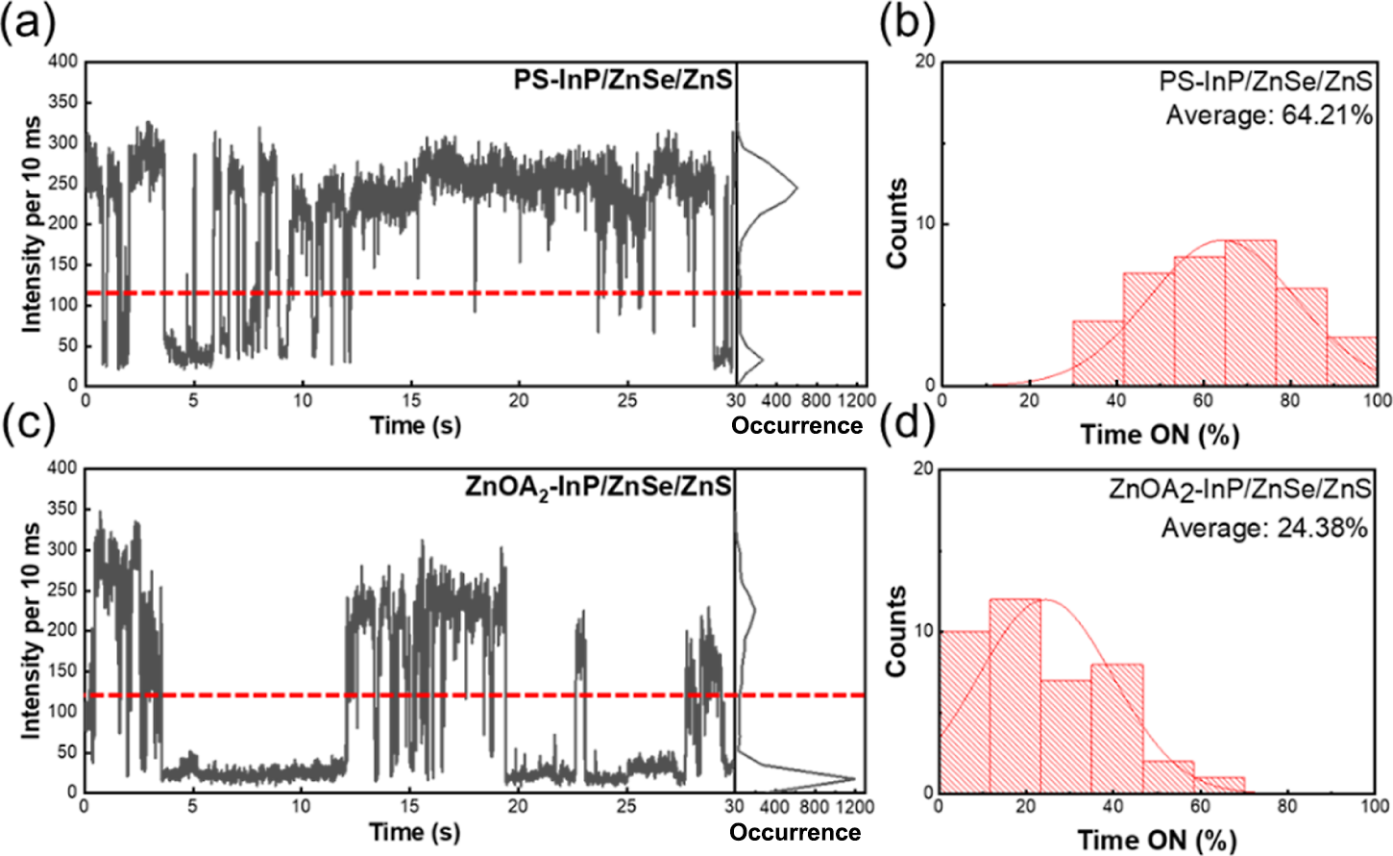
Effect of the ZnS shell thickness caused by dissolution on fluorescence intensity time traces for single InP/ZnSe/ZnS QD. (a,b) PS-InP/ZnSe/ZnS single QD; (a) intensity time trace (left) and occurrence histogram (right). (b) The corresponding ON-time fraction distribution. (c,d) ZnOA_2_-InP/ZnSe/ZnS single QD; (c) Intensity time trace (left) and occurrence histogram (right). (d) The corresponding ON-time fraction distribution.

These blinking observations can be mechanistically linked to chemical defects. This hypothesis is supported by XPS analysis (Figure S7). ZnOA_2_–InP/ZnSe/ZnS exhibits an additional In 3d_5_/_2_ peak at 445.6 eV and a Se–Se doublet near 55–56 eV, indicating the formation of In–S and Se–Se bonding species that are absent in the PS-InP control. These bonding defects introduce midgap trap states that promote nonradiative recombination and are consistent with the observed blinking behavior. Such structural defects ultimately contribute to the lower PLQY (∼64%) measured for ZnOA_2_–InP/ZnSe/ZnS, in contrast to the more stable emission and 92% PLQY observed in the preshelled PS-InP sample. These trap states likely originate from nonuniform shell growth and incomplete passivation induced by core dissolution, which disrupts the shelling process and increases the probability of forming In–S and Se–Se bonding defects. Such defects introduce localized electronic states that act as traps, thereby enhancing nonradiative recombination pathways and blinking.

Figure 7a illustrates variations of the quantity of QDs obtained from two shell growth strategies. In the DI strategy, overcoating of ZnSe preshell can effectively prevent the InP cores from digestion in the heating process, resulting in the particle number yield of InP core–shell QDs being closed to the original InP QDs. In the TI strategy, the final quantity of QDs is only one-third of the original cores, indicating that the digestion phenomenon leads to the two-thirds reduction of the InP core. In contrast, the weight-based production yields of both samples prior to purification are quite similar, as shown in Figure 7b. This apparent similarity arises because the digested sample contains relatively larger-volume QDs, whose increased individual mass compensates for the reduced particle number in the overall sample weight. As previously stated, our earlier studies exhibited that final QD products, despite core digestion, could still achieve high PLQY of nearly 100% due to neglect of low-PLQY particles in dilute or purified samples. However, the overall production yield of high-PLQY QDs in such cases tends to be low. Although optimized shell growth can compensate for some core loss, core digestion introduces variability that is difficult to control in practice. Therefore, we advocate using the DI strategy for synthesizing InP-based QDs: initiating shell growth with a pulse preinjection of monomers during the temperature ramping phase (150–280 °C) to prevent core digestion, followed by a continuous monomer injection at higher temperatures for shell thickening. Finally, a continuous injection of the TOP-S monomer is employed to complete the outer ZnS shell formation. Additionally, introducing InP cores into the shell reactor at temperatures above 280 °C should be avoided as this could accelerate core dissolution.

**Figure 7:**
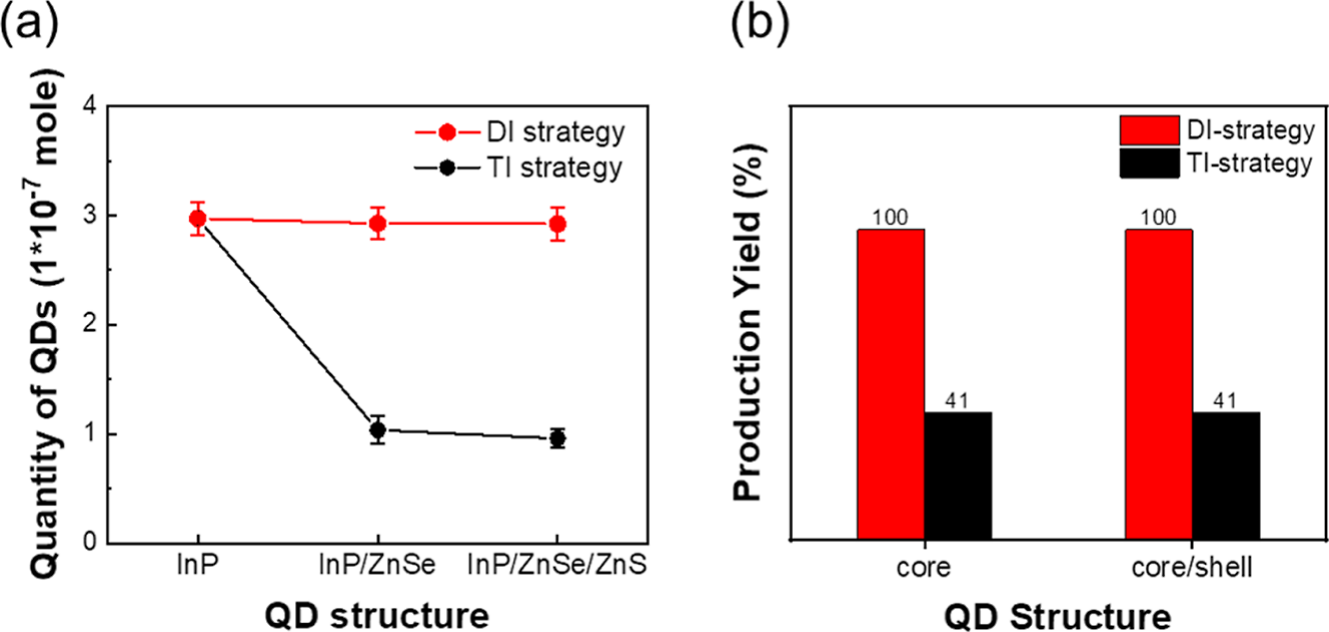
Comparison of particle number and yield of core/shell QDs. (a) Estimated particle quantity at different synthesis stages. (b) Production yield calculated from sample weights.

While the present study focuses on the early stage preservation of InP core integrity during the shelling process, it provides a critical foundation for the successful formation of uniform thick-shell QDs. In our prior work, we demonstrated that controlling shell composition and morphology enables the fabrication of >20 nm InP/ZnSeS/ZnS QDs with high PLQY and enhanced thermal stability.^18^ These two studies address distinct yet complementary aspects of InP QD engineering. The present work focuses on early stage core preservation, while our previous work centers on a strain-relieving shell architecture for thick-shell stability.

## Conclusion

In summary, we uncover facet-selective core digestion as a critical factor limiting the yield and size uniformity of the InP QDs. By introducing a preshell protection step, the InP cores are preserved during the ramping stage, enabling uniform ZnSe/ZnS shell growth. This approach suppresses particle loss, narrows the size distribution, and enhances the experimental reproducibility. Our findings not only clarify a hidden degradation pathway in InP synthesis but also establish a practical method for producing high-quality, cadmium-free QDs at scale for future display technologies.

## Supplementary Material



## Notes

Additional experimental details, including XPS spectra, EDS data, TEM images, and calculation method of QD quantity (PDF)
