# Supplementary material for: Pre-Shell Protection Suppresses Facet-Selective Core Digestion in InP Quantum Dots for High-Yield and Uniform Core/Shell Structures
Source: ACS Appl Mater Interfaces. 2025 Oct 22;17(44):61509–18. doi: 10.1021/acsami.5c16396 (PMC12598700; doi:10.1021/acsami.5c16396)
Supplement: Supplementary file 1 [file am5c16396_si_001.pdf]

Supporting Information

Pre-Shell Protection Suppresses Facet-Selective Core Digestion in InP Quantum Dots for High-Yield and Uniform Core/Shell Structures

You-Cheng Wu,[1] *Hsuan-Yu Lee,*[1] *and Hsueh-Shih Chen*[1,2,3]*

[1]Department of Materials Science and Engineering, National Tsing Hua University, Hsinchu 30013, Taiwan

[2]College of Semiconductor Research, National Tsing Hua University, Hsinchu 30013, Taiwan

[3]Department of Chemical Engineering & Materials Science, College of Engineering, Yuan Ze University, Taoyuan 32003, Taiwan

*Email: chenhs@mx.nthu.edu.tw

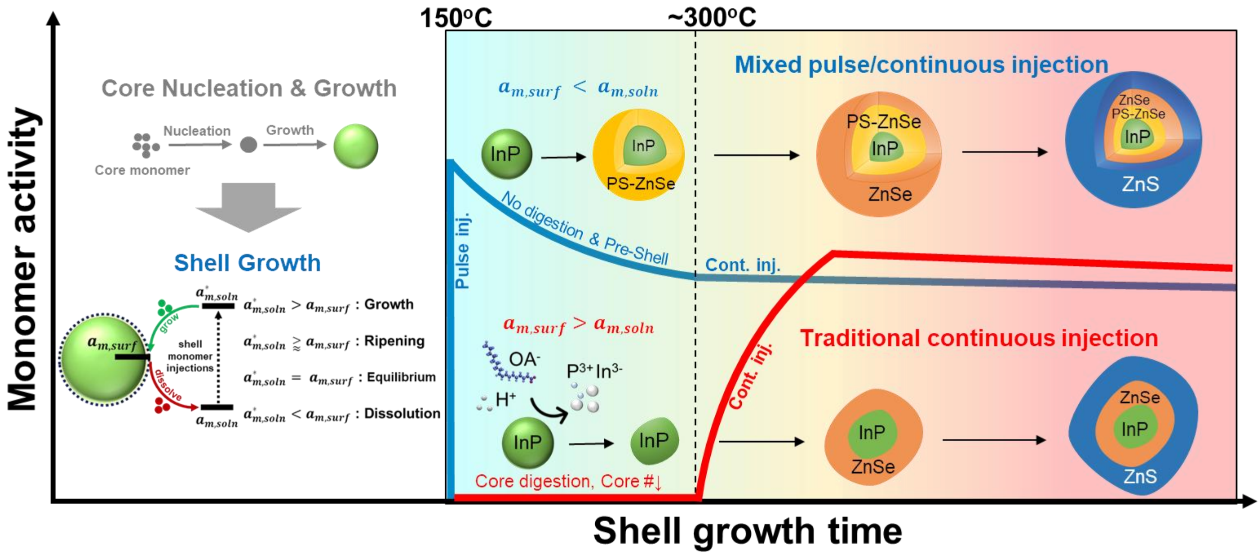

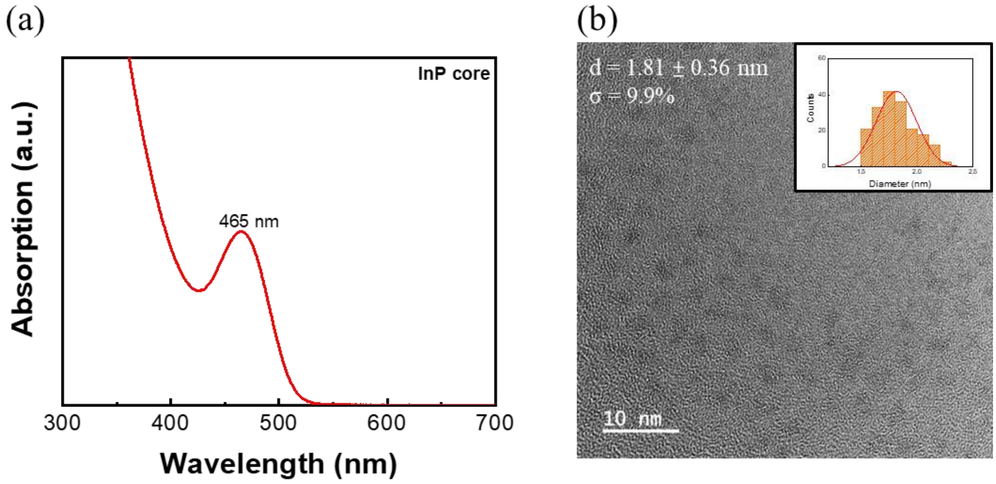

Figure S1. (a) UV-vis spectra of as-prepared InP core. The first exciton absorption peak is 465 nm. (b) TEM image and size distribution histogram (top-right) of InP core, indicating core size is approximately 1.8 nm.

Calculation of QD quantity (particle number/concentration)

To calculate the quantity of QDs, the Beer-Lamber equation was used. In the Beer-Lambert equation: $A = \varepsilon * L * C$, where A is absorbance of absorption peak, ε is intrinsic absorption coefficient of InP QDs ($L*mol^{-1}*cm^{-1}$), L is optical length (cm), and C is molar concentration ($mole*L^{-1}$). By the absorbance of the InP absorption peak in the UV-vis spectra and the intrinsic absorption coefficient, the molar concentration of InP QDs in the quartz cell of UV-vis measurement can be calculated. Through the concentration of InP cores in the quartz cell, the particle number of InP cores in the solution during the synthesis process can be further determined by the equation: Mole of QDs = $0.003*C*V*v^{-1}$, where C ($mole*L^{-1}$) is molar concentration calculated from Beer-Lambert equation, V is volume of InP QDs crude solution (L), and v is volume of InP QDs crude solution for measurement (L). With this method, the particle number of InP cores in the solution can be calculated. In addition, the particle number variation was monitored by tracking the absorbance at the *initial* first excitonic absorption peak wavelength of InP QDs. This peak was adopted to ensure that the particle number change reflects the same size population, as shifting to the red-shifted peak position during digestion could involve other size groups. In addition, in the OA/ODE system, the excitonic peak became indistinct after digestion, preventing reliable reassignment. For consistency and objective comparison among the three reaction conditions (OA/ODE, $Zn(OA)_2$/ODE, and ODE), the initial peak wavelength was therefore used. The effect of *size-dependent* extinction coefficients calculated by absorption *after digestion* was also evaluated, and the resulting particle number trend is similar (provided below for reference).

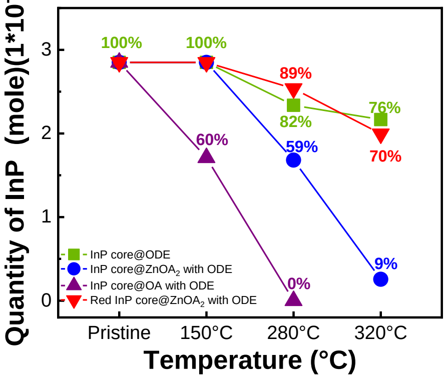

Figure S2. Variation of particle number calculated by size-dependent extinction coefficients corrected by TEM.

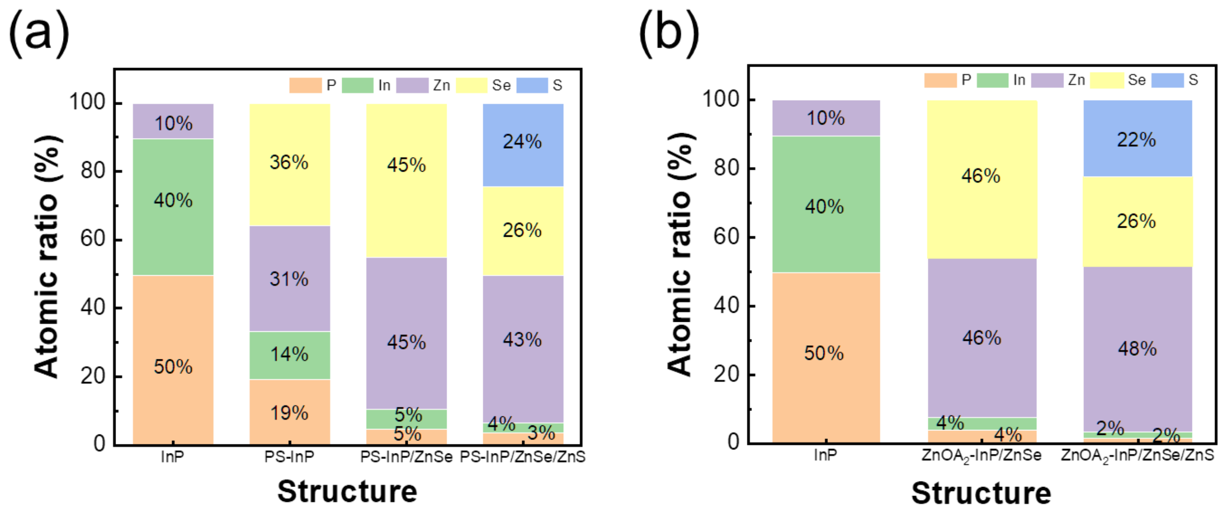

Figure S3. EDS analysis of InP core-shell QDs during the shell growth process in (a) dual injection (DI) strategy and (b) traditional injection (TI) strategy. The chemical ratio of Zn, Se and S gradually increases with shell growth. The Se/S ratio of InP/ZnSe/ZnS in both injection methods is similar ~ 1/1, indicating the core digestion did not significantly influence the final composition of QD products.

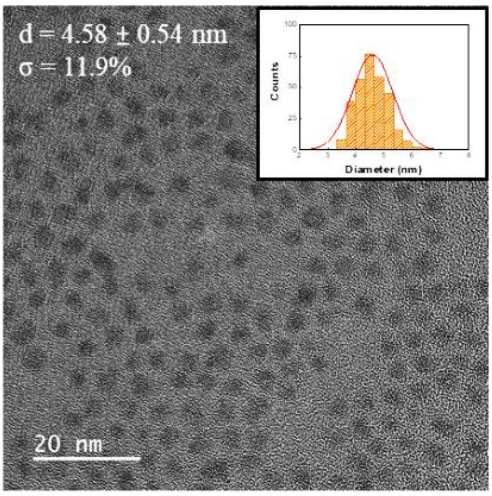

Figure S4. TEM image and size distribution histogram (top-right) of PS-InP QD. The average size of PS-InP is estimated ~ 4.6 nm, indicating that the thickness of ZnSe preliminary shell is 2.8 nm (core size ~ 1.8 nm, shown in **Figure S1**). Prevention of core dissolution by a pulse injection of shell monomers during the temperature ramping stage. The monomer pre-injection leads to some thin shell growth, which is named to be preliminary shell or pre-shell.

Table S1. Fitting parameters for time-resolved PL (TRPL) spectra of PS-InP/ZnSe/ZnS and $ZnOA_2$-InP/ZnSe/ZnS.

Sample	A_1	τ_1 (ns)	A_2	τ_2 (ns)	A_3	τ_3 (ns)	Weighted τ_{av} (ns)
PS-InP/ZnSe/ZnS	0.09	8.26	0.79	30.46	0.12	116.55	61.80
$ZnOA_2$-InP/ZnSe/ZnS	0.15	8.16	0.73	28.32	0.12	103.05	55.46

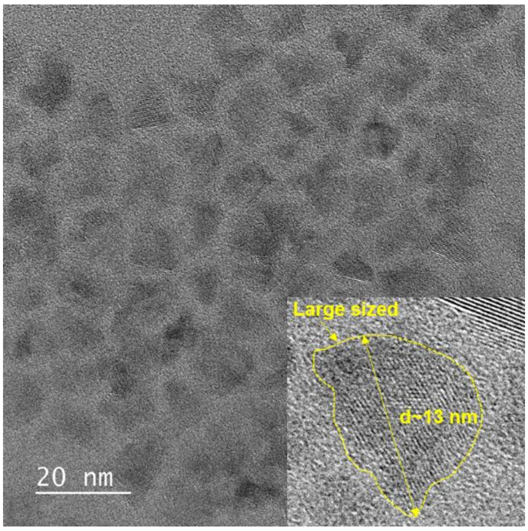

Figure S5. TEM image of large QDs grown without pre-shell $ZnOA_2$-InP/ZnSe/ZnS. Larger particles with lower PLQY were found in the sample, which could be readily removed by washing process. After removing the larger particles, the remained QDs can exhibit high PLQY more than 90%, though the weight yield decreases.

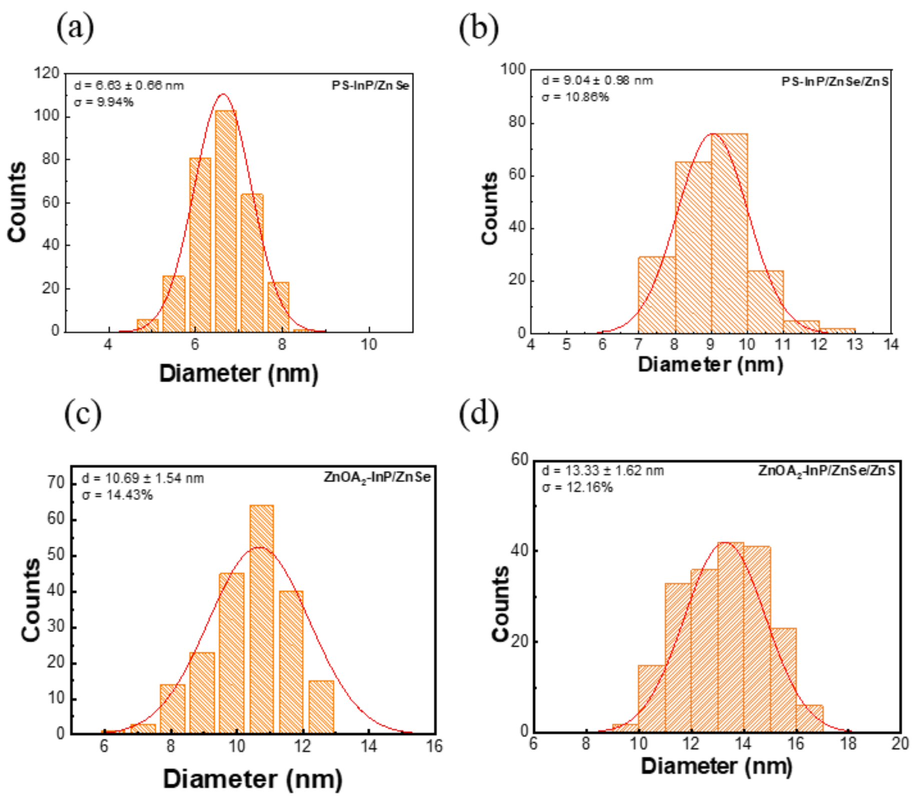

Figure S6. The size histogram shows the average size of (a) PS-InP/ZnSe and (b) PS-InP/ZnSe/ZnS are 6.6 nm ± 0.7 nm and 9.0 ± 1.0 nm. The σ are 9.9% and 10.9% respectively. (c), (d) QD grown without pre-shell $ZnOA_2$-InP/ZnSe and $ZnOA_2$-InP/ZnSe/ZnS are 10.7 nm ± 1.5 nm and 13.3 ± 1.6 nm. The σ are 14.4% and 12.2% respectively, which is calculated by TEM image.

X-ray photoelectron spectroscopy (XPS)

XPS analysis is conducted for PS-InP/ZnSe/ZnS and $ZnOA_2$-InP/ZnSe/ZnS to understand the elemental bonding condition within the core-shell structures. In the In 3d spectra, it can be observed that both PS-InP/ZnSe/ZnS and $ZnOA_2$-InP/ZnSe/ZnS exhibit dual peaks at 444.6 and 452.2 eV, representing In-P bonding within the InP core. However, $ZnOA_2$-InP/ZnSe/ZnS shows an additional $3d_{5/2}$ peak at 445.6 eV, indicating the presence of In-S bonding. In a typical InP/ZnSe/ZnS core-shell structure, the ZnSe is followed by the epitaxial growth of the ZnS shell. In-S bonding should not exist in the InP/ZnSe/ZnS core shell structure. But when InP core undergo digestion before the shell growth, the In ions are presented in the solution. During the subsequent shell growth, these In ions would react with the shell sulfur precursor, resulting in the formation of In-S bonding. The presence of In-S bonding in the shell would influence the epitaxial growth of the ZnS shell and leads to more defects (**Figure S7a**). This aligns with the lower PLQY observed in $ZnOA_2$-InP/ZnSe/ZnS and the higher proportion of non-radiative recombination in PL decay. In **Figure S7b**, both samples exhibit dual peaks at 159.8 and 165.8 eV, which correspond to Zn-Se bonding of ZnSe shell. Additionally, peaks at 161.5 and 162.7 eV represents Zn-S bonding in the ZnS shell. $ZnOA_2$-InP/ZnSe/ZnS also shows a $3p_{1/2}$ peak at 162.2 eV, indicating In-S bonding. However, this peak is less prominent due to overlap with other peaks. In **Figure S7c**, the Se 3d spectra shows dual peaks at 53.9 and 54.7 eV for both samples, representing Zn-Se bonding in the ZnSe shell. However $ZnOA_2$-InP/ZnSe/ZnS additionally exhibits Se-Se bonding peaks at 55.2 and 56.1 eV, indicating the presence of Se-Se aggregation. This might result from excessively fast shell growth in the TI strategy, leading the aggregation of Se element. In the **Figure S7d**, $ZnOA_2$-InP/ZnSe/ZnS shows an additional peak at 1021.1 eV in Zn 2p spectra, indicating the formation of $ZnSO_4$. This might result from oxidation during the sampling process under an atmospheric environment. From the XPS analysis, it is evident that digestion phenomenon influences the elemental bonding. These bonds, such as In-S bonding in the ZnS shell or Se-Se aggregation in the ZnSe shell, would lead to more defect existing in the core shell structure, resulting in poor optical performance of $ZnOA_2$-InP/ZnSe/ZnS.

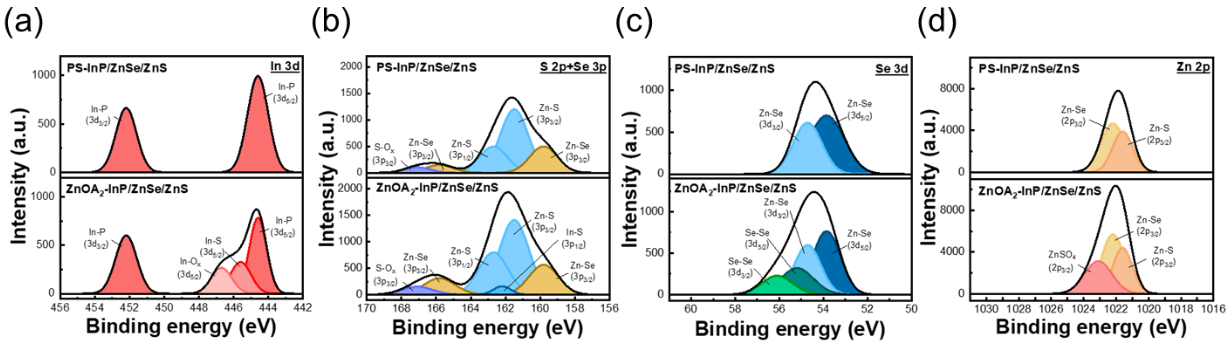

Figure S7. XPS spectra of PS-InP/ZnSe/ZnS and $ZnOA_2$-InP/ZnSe/ZnS QDs. (a) In 3d, (b) S 2p+Se 3p, (c) Se 3d and (d) Zn $2p_{3/2}$.
